# Preparation and Release of pH-Sensitive β-Cyclodextrin Derivative Micelles Loaded with Paclitaxel

**DOI:** 10.3390/polym14122482

**Published:** 2022-06-18

**Authors:** Meirong Zhao, Weiwei Jiang, Xinrong Xie, Yogini Jaiswal, Leonard Williams, Mei Wei, Ying Mo, Yifu Guan, Hua Yang

**Affiliations:** 1 School of Chemistry and Chemical Engineering, Guangxi Minzu University, Nanning 530006, China; zhaomeirong124@163.com (M.Z.); xiexinrong297@163.com (X.X.); 2 Department of Pharmacy, Guangxi Agricultural Vocational University, Nanning 530021, China; 18778141942@163.com (M.W.); morningzai@163.com (Y.M.); 3 College of Medicine, Guangxi University, Nanning 530004, China; thanks202206@163.com; 4 Center for Excellence in Post-Harvest Technologies, North Carolina A&T State University, The North Carolina Research Campus, 500 Laureate Way, Kannapolis, NC 2802, USA; ysjaiswa@ncat.edu (Y.J.); llw@ncat.edu (L.W.)

**Keywords:** polymer micelles, β-cyclodextrin, pH-sensitive, paclitaxel, release

## Abstract

In this paper, a new amphiphilic mono-6-β-cyclodextrin octadecylimine (6-β-CD-N-ODMA) copolymer was synthesized based on β-cyclodextrin and octadecylamine, which can self-assemble to form polymeric micelles. Drug-loaded micelles (a new drug carrier) were obtained using 6-β-CD-N-ODMA and paclitaxel (PTX) by the dialysis method. Orthogonal experiments were used to optimize the preparation method of the drug-loaded micelles. The drug-loading content of the carrier prepared by the optimized method was 1.97%. The physicochemical properties of blank micelles and drug-loaded micelles were evaluated by the fluorescence probe method, infrared spectra, dynamic light scattering, and scanning electron microscopy. The release properties of the carrier were investigated. The carrier has good pH sensitivity and the cumulative release rate after 96 h was 88% in PBS (pH = 5.0). The Ritger–Peppas equation is the optimal model for PTX released at pH 5.0, implying that the hydrolysis effect of 6-β-CD-N-ODMA is the main reason for PTX release. The results indicate that the developed carrier can increase the solubility of PTX and possess potential for increased clinical efficacy of PTX.

## 1. Introduction

In the past few decades, polymeric micelles self-assembled from amphiphilic copolymers have received considerable research interest. They have been successfully used as one of the most promising nanocarriers for anticancer drugs [1,2,3,4,5,6]. Interest of researchers in drug-loaded polymeric nanoparticles has increased due to their many superior properties compared with low molecular weight drugs [3]. Some of these properties include increased circulation time [3,7], reduced kidney clearance [8], protection from enzymatic degradation [4], enhanced solubility of poorly soluble drugs [9], and reduced drug side effects [2].

Cyclodextrins (CDs) are a series of natural macrocyclic oligosaccharides with a hydrophilic exterior and a hydrophobic interior cavity [10,11]. Due to the extremely low toxicity, excellent biocompatibility, and appropriate complexation with a variety of hydrophobic molecules, CDs are the most widely employed host units to construct polymeric micelles for biomedical applications [12,13,14,15,16,17,18]. For example, Poudel et al. prepared a supramolecular hydrogel system by the host–guest interaction between the α-cyclodextrin (α-CD) and poly (ethylene glycol) (PEG) chains of the poly (ethylene glycol)-block-poly (lactic acid) (PEG-b-PLA) micelles [19]. Ramesh et al. synthesized a series of β-CD amphiphilic star-shaped copolymers with exceptional characteristics and their potential as carriers for micelles drug delivery was investigated [20]. However, very few studies report development of amphiphilic copolymer-based drug-delivery carriers using β-CD derivatives/aliphatic amines as block polymers, especially those with β-CD Schiff base derivatives. 

In recent years, many studies report the development of polymeric micelles for enhancing the efficiency of drug delivery in cancer cells [21,22,23]. The pH of extracellular (pH 6.5 to 7.2) and intracellular (pH 5.0 to 6.5) fluids of tumor cells varies significantly from normal cells (pH 7.4) [24,25]. Anti-cancer drugs such as paclitaxel (PTX) and doxorubicin (DOX) exert their anticancer activity inside the tumor cell environment, and hence it is important to consider the pH-sensitivity characteristics in the development of drug-delivery systems [7]. Some functional groups, such as imine, hydrazone, or acetal bonds, can be introduced into the polymer structures to achieve specific pH sensitivity because these functional groups were degraded at low pH values [25,26]. 

PTX is a natural product, isolated from the bark of western *taxus brevifolia*. It is a clinically widely used drug for therapy and treatment of prostate cancer, ovarian cancer, breast cancer, and AIDS-related Kaposi’s sarcoma [27,28]. The use of PTX has been reported to be associated with an undesirable toxicity profile, poor solubility in water, and nontarget-specific nature, which ultimately limit its therapeutic efficacy. Due to these factors, the development of a drug carrier that increases the solubility of PTX and reduces the toxic side effects has been a topic of interest in the field of hydrophobic drug-delivery systems.

In this study, 6-β-DN-ODMA, an amphiphilic copolymer, was designed and synthesized based on β-CD, which can be self-assembled to form micelles. Its performance as a drug-carrier was investigated with paclitaxel as a model drug. The conditions for PTX/6-β-DN-ODMA micelles’ preparation were optimized by the use of orthogonal experiments. Further, the physicochemical and release properties of the carrier were studied.

## 2. Materials and Methods

### 2.1. Reagents and Equipment

N-octadecylamine, *p*-toluenesulfonyl chloride (TsCl), sodium azide, pyrene, and triphenyl phosphorus were purchased from Saen Chemical Technology Co., Ltd., Shanghai, China. Β-cyclodextrin (β-CD) was purchased from China Sinopharm Group., Shanghai, China. Sodium hydroxide, disodium phosphate, sodium dihydrogen phosphate, citric acid, Tween 80, methyl alcohol, and absolute ethanol were purchased from Guangdong Shantou Xilong Chemical Co., Ltd., Shantou, China. Ethylenediamine, isopropanol, and ammonia were purchased from Nanning Lantian Instrument Equipment Co., Ltd., Nanning, China. Paclitaxel (PTX) was purchased from Chengdu Mansite Standard Products Co., Ltd., Chengdu, China. Dimethylformamide (DMF) was purchased from Chengdu Cologne Chemical Reagent Factory.

NMR spectra were recorded on a Bruker Avance-III 600 MHz spectrometer. Chemical shifts (*δ*) were expressed in ppm with reference to the solvent signals and coupling constants (*J*) were reported in Hz. All NMR experiments were performed using standard pulse sequences supplied by the vendor. Fourier transform infrared spectrum (FTIR) was collected in the range of 4000–500 cm^−1^ by an infrared spectrometer (FTIR-8400S, Shimadzu Corporation, Tokyo, Japan). Size distribution and Zeta potential data were monitored using the Zeta-sizer Nano ZS90x (Malvern Instruments Ltd., Worcs, UK). The morphology of synthesized micelles was visualized using scanning electron microscopy (SEM) (SU8020, Hitachi, Tokyo, Japan) at an acceleration voltage of 20 kv.

### 2.2. Synthesis and Characterization of 6-β-CD-N-ODMA

#### 2.2.1. Synthesis of 6-OTs-β-CD 

**Scheme 1:**
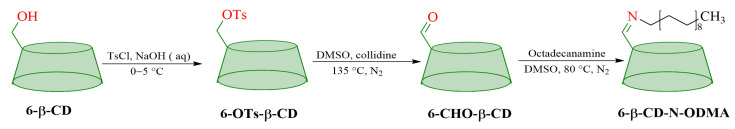
Synthetic route of 6-β-CD-N-ODMA.

As shown in Scheme 1, 10.00 g of β-CD (8.80 mmol) and 200.00 g of NaOH (0.75 mol/L) solution were added to a 500 mL three-necked flask. Upon dissolution of β-CD, 3.36 g of TsCl (17.60 mmol) was added slowly at 0 °C and the resultant mixture was stirred at 0–5 °C (ice-water bath) for 5 h. The unreacted TsCl was separated from the resultant mixture with a sand core funnel, and the pH of the solution was adjusted to 7 with 10% hydrochloric acid. The adjusted solution was kept refrigerated at 0 °C for 10 h, followed by filtration under suction to obtain the crude product. The crude product was then recrystallized three times with deionized water and dried at 60 °C for 24 h to obtain the target product with high purity (28% yield) [29]. ^1^H NMR (600 MHz, DMSO-*d*_6_) *δ* (ppm): 7.75 (2H, d, *J* = 8.1 Hz), 7.43 (2H, d, *J* = 8.2 Hz),5.73 (m, 14H), 4.91–4.72 (m, 7H), 4.61–4.41 (m, 7H), 4.33 (1H, d, *J* = 10.6 Hz), 4.19 (1H, dd, *J* = 11.3, 6.5 Hz), 3.86–3.15 (m, 31H), 2.43 (s, 3H). ^13^C NMR (150 MHz, DMSO-d_6_) *δ* (ppm): 145.30, 133.10, 130.35, 128.02, 102.68, 102.38, 101.72, 82.10, 81.20, 73.52, 72.52, 69.37, 60.38, 60.26, 59.73, 21.67.

#### 2.2.2. Synthesis of 6-CHO-β-CD 

As indicated in Scheme 1, collidine (4 mL) was added to a solution of 6-OTs-β-CD (2.10 g, 1.55 mmol) in anhydrous dimethyl sulfoxide (20 mL). The resultant solution was heated to 135 °C under nitrogen protection and stirred for 1.5 h. After cooling to room temperature, acetone (200 mL) was added dropwise to the reaction solution with vigorous stirring [30]. The resultant precipitate was collected by filtration and dissolved in a small amount of water. Absolute ethanol (200 mL) was added to the solution dropwise and the formed precipitate was then collected and dried in a vacuum drying oven at 60 °C for 24 h to obtain a pale-yellow solid with a yield of 54%. ^1^H NMR (600 MHz, DMSO-d_6_) δ 9.22 (s, 1H), 6.01 (1H, d, *J* = 3.1 Hz), 5.85–5.63 (m, 7H), 5.60–5.42 (m, 15H), 5.04 (s, 6H), 4.92 (s, 1H), 4.83 (4H, d, *J* = 3.6 Hz), 4.61–4.40 (m, 7H), 3.64 (m, 29H). ^13^C NMR (150 MHz, DMSO) δ 188.03, 102.38, 100.76, 100.41, 81.98, 73.60, 72.84, 72.49, 72.10, 60.76, 60.37, 40.15, 30.90. 

#### 2.2.3. Synthesis of 6-β-CD-N-ODMA 

To a solution of 6-CHO-β-CD (0.50 g, 0.44 mmol) in anhydrous DMSO (10 mL), octadecylamine (0.12 g, 0.44 mmol) was added with constant stirring at room temperature (Scheme 1). The reaction mixture was then stirred at 80 °C for 12 h under the nitrogen protection followed by dropwise addition of acetone (80 mL) with vigorous stirring [31]. The resultant mixture was cooled to room temperature and the precipitate was collected by filtration. The product was vacuum-dried at 50 °C for 72 h (71% yield). ^1^H NMR (600 MHz, DMSO-*d*_6_) δ 5.47(m, 7H), 5.16–4.88 (m, 5H), 4.84 (s, 1H), 4.47 (m, 8H), 3.06 (m, 13H), 1.59–1.36 (m, 2H), 1.24 (s, 26H), 0.86 (3H, t, *J* = 6.9 Hz). ^13^C NMR (150 MHz, DMSO) δ: 102.40, 100.78, 81.99, 79.85, 73.63, 72.86, 72.49, 72.12, 60.77, 60.37, 56.51, 31.75, 29.50, 29.16, 22.56, 19.00, 14.42, 8.20. FT-IR: 3357, 2924, 2853, 1654, 1600, 1415, 1368, 1155, 1080, 1028, 936, 857, 759, 706, 577, 525 cm^−1^.

### 2.3. Preparation of PTX/6-β-CD-N-ODMA Micelles

There are several methods reported for the use of polymer micelles for encapsulation of hydrophobic drugs [32,33]. The critical aspect for this application is the optimization of the loading method to avoid supersaturation, as this in turn could lead to instability and precipitation of dissolved drugs during storage [34]. Based on a detailed understanding of the characteristics of β-CD, a new dialysis method was designed to prepare 6-β-CD-N-ODMA micelles [35].

(1) Preparation of drug-loaded micelles

Drug-loaded micelles were prepared using PTX as a model drug at 25 °C. Briefly, 50 mg of 6-β-CD-N-ODMA was prepared in a 0.12 mg/mL DMF solution, and 3 mg of PTX was dissolved in 5 mL of DMF (0.06 mg/mL). Then, the two solutions were mixed under magnetic stirring. The mixed solution was then added dropwise to 2 mL of deionized water. In the next step, the solution was transferred to a dialysis bag (MWCO 3500, Shanghaiyuanye Bio-Technology Co., Ltd., Shanghai, China) in 2 mL of deionized water. The bag was kept for dialysis and changed for fresh deionized water at specific intervals. After 24 h of dialysis, PTX micelles solution was obtained by microporous membrane filtration, and drug-loaded micelles were obtained after freeze-drying. The blank micelles were prepared by the same method. 

(2) Drug-loading content (DLC%) and encapsulation efficiency (EE%)

The drug-loading content (DLC%) and encapsulation efficiency (EE%) of the prepared micelles were considered as the main evaluation factors for the quality of the synthesized PTX/6-β-CD-N-ODMA micelles. The drug-loading content and encapsulation efficiency of PTX/6-β-CD-N-ODMA micelles were determined as follows: About 10 mg of PTX/6-β-CD-N-ODMA micelles solution was weighed and transferred to a 50 mL volumetric flask, which was filled by methanol. The micelles were destroyed by ultrasonic mixing in a water bath, leading to complete dissolution of PTX in methanol. The concentration of PTX was estimated by UV absorbance at a 227 nm wavelength using methanol as a blank solution. The encapsulation efficiency and drug loading are calculated as in Equations (1) and (2), respectively:

(1) $$\mathrm{EE}\%=\frac{m}{m_{0}}\times 100\%$$

(2) $$\mathrm{DLC}\%=\frac{m}{m+m_{1}}\times 100\%$$

where m is the weight of the drug in micelles, m_0_ is the weight of the drug, and m_1_ is the weight of the drug-carrying material.

#### 2.3.1. Single-Factor Study

The size of the micelles prepared by the dialysis method is affected by four factors: the quantity of organic solvent, polymer, drug, and water.

(1) The influence of the amount of organic solvents

6-β-CD-N-ODMA (40 mg) and PTX (3 mg) were dissolved in specific volumes of DMF (2, 3, 5, 7, 10, and 12 mL). The resultant solution was then added dropwise to 2 mL of deionized water and mixed well. This prepared mixture was then subjected to dialysis in water for 24 h (WMCO 3500) and followed by exchange with fresh deionized water at 2, 4, 6, 8, 12, and 24 h intervals. After 24 h of dialysis, the solution from the dialysis bag was poured into a container and subjected to centrifugation at 3000 r/min for 10 min. The supernatant was filtered with a microporous membrane to remove the uncoated drugs and the micellar solution was placed in a refrigerator at 4 °C.

(2) Effect of amphiphilic polymer 

PTX (3 mg) was mixed with varying quantities of 6-β-CD-N-ODMA (20, 30, 40, 50, 60, and 70 mg) and the resultant mixture was dissolved in 5 mL of DMF. The mixture was added dropwise to 2 mL of deionized water and stirred to obtain a uniform mixture. The mixture was then transferred to a dialysis bag (WMCO 3500), and the same dialysis steps described above were applied to this mixture. 

(3) Influence of hydrophobic drug 

6-β-CD-N-ODMA (40 mg) was added to varying quantities of PTX (1, 2, 3, 4, 5, and 6 mg) and the mixture was dissolved in 5 mL of DMF. The resultant solution was then added dropwise to 2 mL of deionized water with occasional stirring. The solution was then transferred for dialysis (WMCO 3500).

(4) Influence of water 

Here, 40 mg of 6-β-CD-N-ODMA and 3 mg of PTX were dissolved in 5 mL of DMF. The solution was then added dropwise to varying amounts of water (1, 2, 3, 4, 5, and 6 mL) and stirred occasionally. The resultant solution was then dialyzed (WMCO 3500).

#### 2.3.2. Orthogonal Test for Optimization of Drug-Loaded Micelles Preparation

In the single-factor test, the drug loading was selected as the measurement index to investigate the influence of drug dosage, polymer, DMF, and water. The orthogonal table L_16_(4^5^) was used to design 16 sets of experiments (4 factors and 4 levels) for the preparation of drug-loaded micelles (Table 1).

#### 2.3.3. Determination of Critical Micellar Concentration (CMC)

The CMC of the polymer 6-β-CD-N-ODMA was determined by the pyrene fluorescence probe Photometer (PF-5301PC, Shimadzu Corporation, Tokyo, Japan). The ratio of the third emission wavelength and first emission wavelength of pyrene is closely related to the polarity of the solution. With the formation of the micelle, pyrene rapidly transfers from the polar environment to the non-polar environment, resulting in the ratio of I_3_/I_1_. Then, 6-β-CD-N-ODMA (40 mg) was used to prepare a reserve solution of 0.4 mg/mL with deionized water. A polymer solution with a concentration of 4 × 10^−4^−0.4 g/L was prepared. Pyrene-acetone solution (1 mL, 0.04 g/L) and polymer aqueous solution were added to 10 mL of amber-colored volumetric bottles and subjected to ultrasonication by an ultrasonic cleaner (KQ-50B, Kunshan Ultrasonic Instrument Co., Ltd., Kunshan, China) for 30 min at 25 °C. The solutions were kept in the dark overnight. The fluorescence intensity of solutions of each concentration were measured at 394 and 373 nm emission wavelengths by a fluorescence spectrophotometer. 

The logarithm (logC) of each concentration was plotted by the fluorescence intensity ratio (I394/I373), and the critical micelle concentration of the polymer 6-β-CD-N-ODMA was determined.

#### 2.3.4. Properties and Characterization of PTX/6-β-CD-N-ODMA

(1) Fourier transform infrared spectrum 

FTIR spectra for PTX, 6-β-CD-N-ODMA, and the physical mixtures of both PTX/6-β-CD-N-ODMA and PTX/6-β-CD-N-ODMA were obtained in the range of 4000–500 cm^−1^ on a FTIR-8400S spectrometer (32 scans at a resolution of 4 cm^−1^) using KBr pellets.

(2) Particle size distribution and Zeta potential

The particle size and Zeta potential of blank micelles (6-β-CD-N-ODMA) and PTX/6-β-CD-N-ODMA were measured by dynamic light scattering (DLS) at room temperature. The particle size distribution was characterized by the intensity-averaged particle size and polydispersity index (PDI). The 6-β-CD-N-ODMA blank micelle solution and the PTX/6-Β-CD-N-ODMA micelle solution were prepared, respectively. The particle size distribution and Zeta potential of drug-loaded micelles and blank micelles in aqueous solution were measured in triplicates by a 633 nm wavelength at 90 and 25 °C, respectively.

(3) Scanning electron microscopy 

SEM is a qualitative method used to observe the surface morphology of the substance. The SEM of products was obtained with the environmental scanning electron microscope. A few freeze-dried blank micelles (6-β-CD-N-ODMA) and drug-loaded micelles (PTX/6-β-CD-N-ODMA) with a 5 nm platinum coating were observed at the resolution of 1 and 3 K, respectively.

### 2.4. Estimation of Drug Release 

We used the dialysis method to study the drug release of PTX from PTX/6-β-CD-N-ODMA in PBS (pH = 7.4 and pH = 5.0) with 1% Tween 80 as the release medium, to simulate pH conditions in blood circulation and intracellular, respectively. Briefly, 3 mL of PTX/6-β-CD-N-ODMA (PTX, 0.06 mg/mL) was subjected to dialysis (WMCO 3500) at 37 °C in 50 mL of PBS buffer solution containing 1% (*w*/*v*) Tween 80 with constant speed at 120 rpm. At the scheduled time intervals, 4 mL of external release medium was taken out for analysis and an equal volume of fresh release medium was added simultaneously into the release system. Finally, the determination of the drug concentration was carried out by a UV spectrophotometer. The cumulative drug release (E_r_) was calculated using the following formula:

(3) $$E_{r}=\frac{\sum_{i=1}^{n}V_{0}\times C_{t}}{W_{0}}\times 100\%$$

where *V*_0_ is the volume of the release medium, *C*_t_ is the concentration of the drug in the release medium at each time point, and W_0_ is the measured active ingredient content in the drug-loaded micelles used for the study of release. 

## 3. Results and Discussion

### 3.1. Synthesis of 6-β-CD-N-ODMA

The synthesis of the 6-β-CD-N-ODMA was accomplished using a three-step procedure starting with β-CD (Scheme 1). The first step of the synthesis involved the well-known reaction of β-CD with TsCl in aqueous NaOH solution to form the monotosylate (6-OTs-β-CD). The selectivity of the tosylation was accomplished by a heterogeneous reaction at low temperature. The purified tosylate was then converted to the aldehyde (6-CHO-β-CD) via the Nace reaction. This oxidation was performed by heating the tosylate in DMSO with collide added as a non-nucleophilic base. Then, aldehyde, 6-CHO-β-CD, was introduced in the reaction with octadecylamine, leading to the corresponding imine in 71% yield.

### 3.2. Critical Micelle Concentration of 6-β-CD-N-ODMA

**Figure 1:**
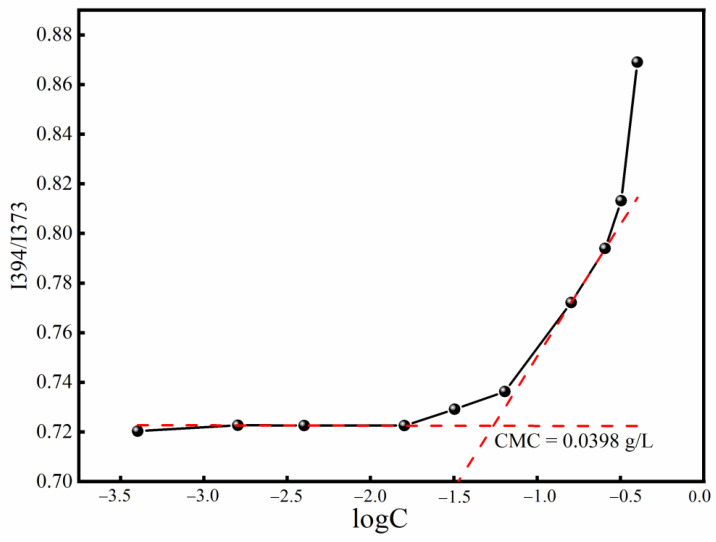
Variation of intensity ratio (I394/I373) versus logarithm of 6-β-CD-N-ODMA concentration.

The logarithmic representation of the critical micelle concentration (CMC) of the polymer 6-β-CD-N-ODMA is shown in Figure 1. The calculated CMC is 3.98 × 10^−2^ g/L. The micelle formed by the β-cyclodextrin polymer (6-β-CD-N-ODMA) has better thermodynamic stability in aqueous solution compared to the concentrations commonly used with lower molecular weight surfactants. This characteristic keeps the micelle structure intact when diluted to a lower concentration.

### 3.3. Formulation Optimization of PTX/6-β-CD-N-ODMA

#### 3.3.1. Standard Curve of PTX

**Figure 2:**
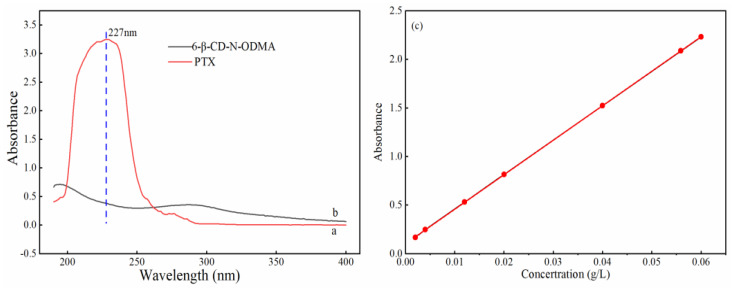
The UV-vis spectra of PTX (**a**), 6-β-CD-N-ODMA (**b**), and the standard curve of PTX (**c**).

The UV-vis spectra of the sample and standard solutions (a: PTX, b: 6-β-D-N-ODMA) are shown in Figure 2. The methanol solution of PTX (0.02 mg/mL) has maximum absorption at 227 nm (Figure 2a), while the absorption value at 227 nm of 6-β-CD-N-ODMA is lower (Figure 2b). Therefore, 6-β-CD-N-ODMA does not interfere with the determinations of the drug-loading test. The PTX standard linearity curve regression equation was found to be y = 35.419x + 0.1035, R^2^ = 0.99998 (Figure 2c). The results indicate that the PTX standard is linear in the concentration of 2−60 mg/L, and this value can be used in the determination of drug-loading micelles. The average recovery rate of low-, medium-, and high-concentration solutions of PTX is 99.19%, and RSD is 0.462%.

#### 3.3.2. Single-Factor Analysis

When PTX/6-β-CD-N-ODMA was prepared by the dialysis method, the obtained PTX-loading micelle was affected by the quantity of the organic solvent, the drug, and water, and the dosage of 6-β-CD-N-ODMA. The results of the effect of these factors are shown in Figure S1.

The drug loading and encapsulation efficiency of micelles reached a maximum of 1.79% and 72.9% with 3 mL of DMF. Interestingly, the drug loading and encapsulation efficiency of micelles decreased with the increase of DMF over 3 mL (Figure S1a). The results suggest that the drug and material in the solvent to disperse is unfavorable to the drug package due to excess solvent. The encapsulation efficiency and drug loading decreased with the increase of PTX and reached the maximum of 1.8% and 73.5% with 1 mg of PTX (Figure S1b). The encapsulation efficiency of micelles increases with the increase of polymer dosage, whereas the drug loading increases first and then decreases. The drug loading of micelles reached the maximum of 1.7% with 40 mg of polymer (Figure S1c). The encapsulation efficiency increased first and then decreased with the increase of the water consumption value. The encapsulation efficiency of micelles reached the maximum of 67.3% with 3 mL of water. The drug loading does not show a significant increase with the increase of water consumption values (Figure S1d).

#### 3.3.3. Orthogonal Result Analysis

Orthogonal experiments were carried out to optimize the DMF volume, 6-β-CD-N-ODMA and PTX quantities, and the volume of water consumption. The orthogonal design and results are indicated in Table 2. It can be concluded that the effects of various factors on the drug loading follow the order of: C > B > A > D, by extreme difference analysis (R) (Table 3). After optimization of experimental conditions, the best condition (A_3_B_3_C_2_D_2_) for the PTX-loading process was found to be with 3 mg of PTX, 50 mg of 6-β-CD-N-ODMA, 5 mL of DMF, and 2 mL of water. 

Parallel experiments were carried out three times to prepare PTX/6-β-CD-N-ODMA based on the optimized formulation. The drug loading of PTX was determined as 1.97% ± 0.12% *(n* = 3). These results indicate that the optimized conditions are stable and reliable.

### 3.4. Characterization of PTX/6-β-CD-N-ODMA

#### 3.4.1. FTIR Analysis

**Figure 3:**
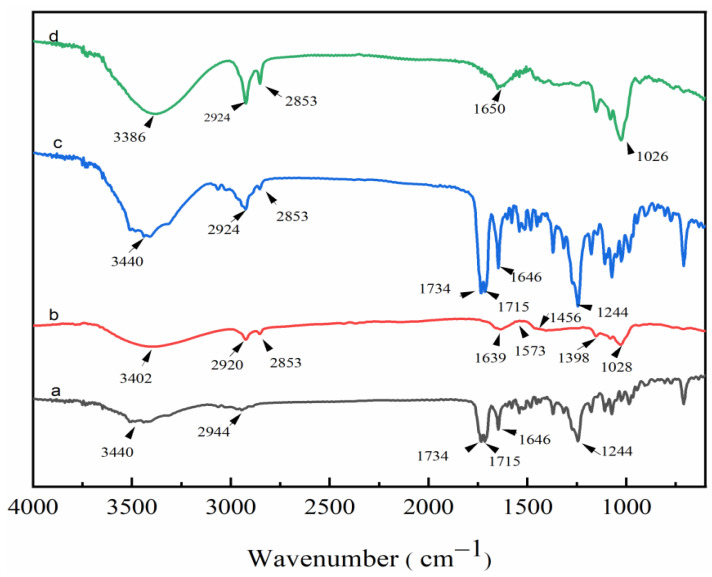
FTIR spectra of PTX (**a**), 6-β-CD-N-ODMA (**b**), mixture (PTX and 6-β-CD-N-ODMA) (**c**), and PTX/6-β-CD-N-ODMA (**d**).

The results obtained from FTIR spectrum analysis were used for confirmation of the formation of the PTX/6-β-CD-N-ODMA, as indicated in Figure 3. The FTIR spectra of PTX (a) is characterized by the prominent band at 3440 cm^−1^ for the O-H stretching vibrations and 2944 cm^−1^ for the C-H stretching vibrations. There was a very strong absorption peak at 1734 and 1715 cm^−1^ for C=O stretching vibration, as well as by the 1646 cm^−1^ bands for C=O stretching vibrations in the amide group. The FTIR spectrum of PTX was characterized by dense peaks in the fingerprint region. The FTIR spectrum of 6-β-CD-N-ODMA (b) displays characteristic absorption peak at 2853 cm^−1^ for the C-H stretching vibrations, 1639 cm^−1^ for C=O stretching vibration, a band at 1456 cm^−1^ corresponding to the CH_3_ stretching vibrations, and at 1398 cm^−1^ for the CH_2_ flexural vibration. Additionally, the absorption band of C=N stretching vibrations was found at 1573 cm^−1^. The spectra of the physical mixtures of both PTX/6-β-CD-N-ODMA (c) corresponded with the superposition of the spectra of the individual components. As for the spectra of the PTX/6-β-CD-N-ODMA (d) in comparison with PTX (a) and the physical mixture (c), the absorption bands at 1734 and 1715 cm^−1^ for C=O stretching vibration from PTX and dense peaks in the fingerprint region of PTX disappeared. The peak of the stretching vibration of O-H strengthened and shifted to a higher wavenumber (from 3440 to 3386 cm^−1^). This suggests that stronger interactions existed between PTX and 6-β-CD-N-ODMA. Compared to the spectrum of 6-β-CD-N-ODMA (b), the peak of the stretching vibration of O-H strengthened and shifted to a higher wavenumber (from 3402 to 3386 cm^−1^). The band at 2920 cm^−1^ corresponding to the stretching vibration of C-H strengthened and shifted to a higher wavenumber (2924 cm^−1^). The band at 1639 cm^−1^ corresponding to the stretching of C=O shifted to a higher wavenumber (1650 cm^−1^). The 1028 and 2853 cm^−1^ bands corresponding to the stretching of C–O and C–H stretching vibrations are quite similar to 6-β-CD-N-ODMA. However, the characteristic absorption peak of PTX is barely visible. Based on these data, we infer that PTX was possibly encapsulated in the core of 6-β-CD-N-ODMA. 

#### 3.4.2. Particle Size Distribution and Zeta Potential Analysis

**Figure 4:**
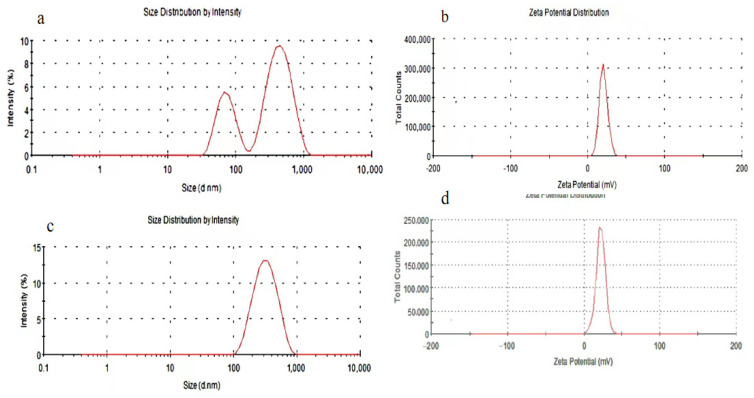
Particle size distribution and Zeta potential distribution of 6-β-CD-N-ODMA (**a**,**b**) and PTX/6-β-CD-N-ODMA (**c**,**d**).

The DLS method was selected to measure the particle size, Zeta potential, and distribution of 6-β-CD-N-ODMA and PTX/6-β-CD-N-ODMA. The particle size distribution and Zeta potential distribution of 6-β-CD-N-ODMA and PTX/6-β-CD-N-ODMA are shown in Figure 4. The particle size distribution of 6-β-CD-N-ODMA shows a bimodal distribution with a wide particle size distribution of 165.7 nm, PDI = 0.571, and ζ = 22.3 mV. The average particle size of PTX/6-β-CD-N-ODMA is about 261.6 nm, PDI = 0.235, and ζ = 20.3 mV. Since the drug is sealed in the hydrophobic core of the micelle, the average particle size of PTX/6-β-CD-N-ODMA is larger compared to 6-β-CD-N-ODMA with a narrow distribution and reduced Zeta potential.

#### 3.4.3. SEM Analysis

**Figure 5:**
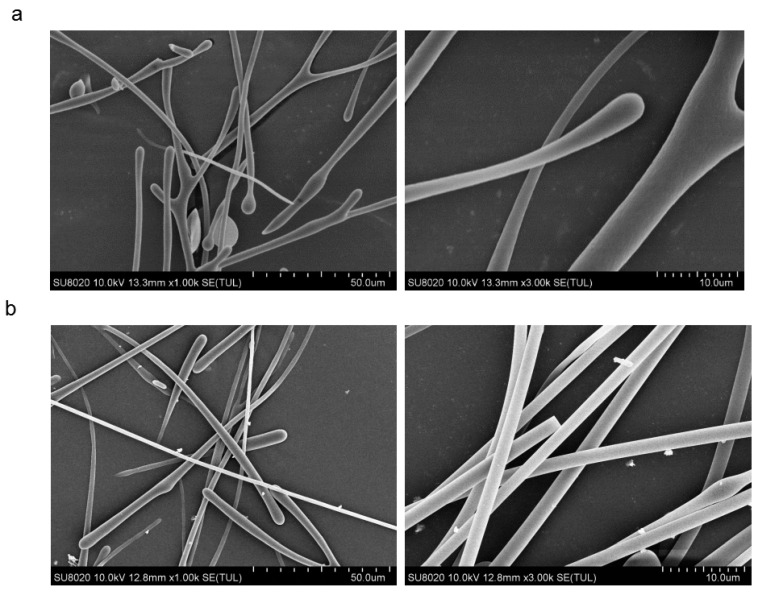
SEM images of 6-β-CD-N-ODMA (**a**) and PTX/6-β-CD-N-ODMA (**b**).

The morphology and topography of the obtained materials were visualized using SEM. The morphology of blank micelles (6-β-CD-N-ODMA) and drug-loaded micelles (PTX/6-β-CD-N-ODMA) was observed by SEM at the resolution of 1 and 3 K, respectively (Figure 5). The 6-β-CD-N-ODMA micelles (a) were rod-shaped, uneven-shaped, and similar to the diameter measured by dynamic light scattering. The morphology analysis of the PTX/6-β-CD-N-ODMA carrier micelle (b) shows the formation of a thick head and a thin tail structure that become gradually uniform with the clustered micelle particles of the cell body.

### 3.5. Sustained Release Behavior of PTX 

**Figure 6:**
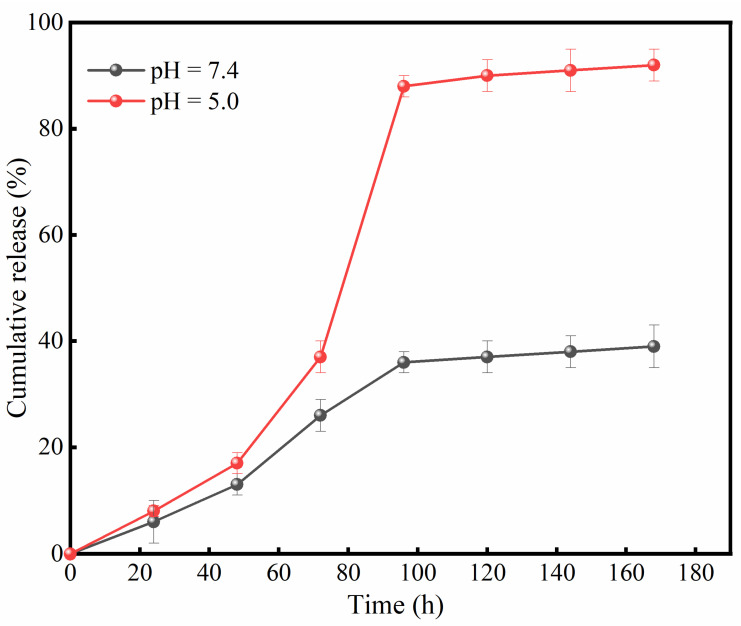
Release of PTX from PTX/6-β-CD-N-ODMA in PBS (pH = 7.4 or 5.0) with 1% Tween 80 at 37 °C.

The drug release at different pH levels of PBS with 1% Tween 80 was studied. The results of the release behavior of the 6-β-CD-N-ODMA-based drug-delivery system in PBS solution at different pH levels are indicated in Figure 6. After 96 h, about 88% of PTX was released in PBS solution at pH = 5.0 and 40% of PTX was released in PBS solution at pH = 7.4. The amount of release of 6-β-CD-N-ODMA polymer micelles was greatly affected by environmental acidity. Such fast release of PTX in weak acid environments was probably because of the hydrolysis of the imine bonds on 6-β-CD-N-ODMA, and this facilitates the release of PTX from the polymer micelles. 

To further explore the release mechanism of PTX, the different kinetic models of PTX released from PTX/6-β-CD-N-ODMA were discussed. As shown in Table 4, the kinetics of PTX released from PTX/6-β-CD-N-ODMA at pH 5.0 was compatible with the Ritger–Peppas equation, and the release coefficient of n (1.403) ˃ 0.85 was consistent with the release mechanism for PTX being determined by synergy between the hydrolysis effect of 6-β-CD-N-ODMA polymer micelles and Fick’s second law of diffusion [36]. These results implied that PTX/6-β-CD-N-ODMA presented a pH-responsive release performance.

## 4. Conclusions

In the present research work, a new amphiphilic copolymer was synthesized by three steps using β-CD as the starting material. pH-sensitive micelles based on the amphiphilic copolymer were developed and studied as hydrophobic, anticancer drug nanocarriers. The formulation of micelles was successfully achieved, and the physicochemical characteristics were confirmed. The designed carrier exhibited good pH sensitivity and a cumulative release rate in PBS. The release kinetics showed that PTX release from PTX/6-β-CD-N-ODMA at pH 5.0 was consistent with the Ritger–Peppas equation. The release coefficient of n showed that the hydrolysis effect of 6-β-CD-N-ODMA polymer micelles and Fick’s second law of diffusion were major release mechanisms. The results of our study suggest that the developed polymeric micelles have potential for increasing the clinical efficacy of PTX. The study provides a basis for future studies in other biological models that can explore the potential pharmaceutical and clinical uses of PTX drug carriers.

## Figures and Tables

**Table 1:** Factors and levels for orthogonal tests.

Level	PTX/mg A	6-β-CD-N-ODMA/mg B	DMF/mL C	Water/mL D
1	1	30	3	1
2	2	40	5	2
3	3	50	7	3
4	4	60	10	4

**Table 2:** L_16_(4^5^) orthogonal design table and results (*n* = 3).

No.	A	B	C	D	Blank	Drug Loading (%)
1	1	1	1	1	1	0.51
2	1	2	2	2	2	1.08
3	1	3	3	3	3	0.99
4	1	4	4	4	4	0.66
5	2	1	2	3	4	1.27
6	2	2	1	4	3	1.14
7	2	3	4	1	2	1.03
8	2	4	3	2	1	0.724
9	3	1	3	4	2	0.86
10	3	2	4	3	1	0.62
11	3	3	1	2	4	1.96
12	3	4	2	1	3	1.45
13	4	1	4	2	3	0.91
14	4	2	3	1	4	1.12
15	4	3	2	4	1	1.24
16	4	4	1	3	2	1.06
K1	0.810	0.888	1.167	1.028	0.774	
K2	1.041	0.990	1.260	1.169	1.008	
K3	1.222	1.305	0.923	0.985	1.123	
K4	1.083	0.973	0.805	0.975	1.252	
R	0.412	0.417	0.455	0.194	0.478	

**Table 3:** Results of variance analysis of orthogonal test.

Factors	Sum of Squares of Deviations	Degrees of Freedom	Ratio of F	The Critical Value of F
A	0.352	3	0.710	9.280
B	0.402	3	0.810	9.280
C	0.534	3	1.077	9.280
D	0.096	3	0.194	9.280
Error	0.500	3		

F (3, 3) = 9.28, α = 0.05.

**Table 4:** Representative models used for describing kinetics of PTX released.

pH	Model	Equation	r^2 a^
7.4	Zero-order kinetics	Q_1_ = 6.131t_1_ + 2.917	0.881
	First-order kinetics	ln(100 − Q_1_) = −0.079t_1_ + 4.585	0.895
	Higuhi equation	Q_1_ = 17.358t_1_^1/2^ − 4.869	0.892
	Weibull equation	lnln[1/(1 − Q_1_/100)] = 1.134lnt_1_ − 2.664	0.929
	Ritger–Peppas equation	lnQ_1_ = 1.015ln t_1_ + 1.923	0.922
5.0	Zero-order kinetics	Q_2_ = 15.821t_2_ − 2.5	0.860
	First-order kinetics	ln(100 − Q_2_) = −0.444t_2_ + 4.899	0.850
	Higuhi equation	Q_2_ = 42.887t_2_^1/2^ − 19.376	0.784
	Weibull equation	lnln[1/(1 − Q_2_/100)] = 2.001lnt_2_ − 2.658	0.914
	Ritger–Peppas equation	lnQ_2_ = 1.403lnt_2_ + 2.082	0.925

^a^ Regression factors.

## Data Availability

The data presented in this study are available on request from the corresponding author.

## Supplementary Materials

The following are av available online at https://www.mdpi.com/article/10.3390/polym14122482/s1, Figure S1: Results of the effect of the quantity of DMF (a), PTX (b), polymer (c), and water (d) on PTX loading; Figure S2: ^1^H NMR of 6-OTs-β-CD; Figure S3: ^13^C NMR of 6-OTs-β-CD; Figure S4: ^1^H NMR of 6-CHO-β-CD; Figure S5: ^13^C NMR of 6-CHO-β-CD; Figure S6: ^1^H NMR of β-CD-N-ODMA; Figure S7: ^13^C NMR of β-CD-N-ODMA.

## References

[1] Croy S.R., Kwon G.S. (2006). Polymeric micelles for drug delivery. Curr. Pharm. Des..

[2] Cai M.T., Zhu K., Qiu Y.B., Liu X.R., Chen Y.W., Luo X.L. (2014). pH and redox-responsive mixed micelles for enhanced intracellular drug release. Colloids Surf. B.

[3] Biswas S., Kumari P., Lakhani P.M., Ghosh B. (2016). Recent advances in polymeric micelles for anti-cancer drug delivery. Eur. J. Pharm. Sci..

[4] Pasut G., Veronese F.M. (2009). PEG conjugates in clinical development or use as anticancer agents: An overview. Adv. Drug Del. Rev..

[5] Sun H.L., Guo B.N., Cheng R., Meng F.H., Liu H.Y., Zhong Z.Y. (2009). Biodegradable micelles with sheddable poly (ethylene glycol) shells for triggered intracellular release of doxorubicin. Biomaterials.

[6] Edgar P.H., Alberto F.M. (2015). Advanced targeted therapies in cancer: Drug nanocarriers, the future of chemotherapy. Eur. J. Pharm. Biopharm..

[7] Deng C., Jiang Y.J., Cheng R., Meng F.H., Zhong Z.Y. (2012). Biodegradable polymeric micelles for targeted and controlled anticancer drug delivery: Promises, progress and prospects. Nano Today.

[8] Xu Y., Wang L., Li Y.K., Wang C.Q. (2016). Reduction and pH dual-responsive nanoparticles based chitooligosaccharide-based graft copolymer for doxorubicin delivery. Colloids Surf. A Physicochem. Eng. Asp..

[9] Wang J., Yang G., Guo X., Tang Z.M., Zhong Z.D., Zhou S.B. (2014). Redox-responsive polyanhydride micelles for cancer therapy. Biomaterials.

[10] Zhang J.X., Ma P.X. (2013). Cyclodextrin-based supramolecular systems for drug delivery: Recent progress and future perspective. Adv. Drug Deliv. Rev..

[11] Mura P. (2014). Analytical techniques for characterization of cyclodextrin complexes in the solid state: A review. J. Pharm. Biomed. Anal..

[12] Gaucher G., Dufresne M.H., Sant V.P., Kang N., Maysinger D., Leroux J.C. (2005). Block copolymer micelles: Preparation, characterization and application in drug delivery. J. Control. Release.

[13] Dharmendra N., Bhattarai J.K., Demchenko A.V., Stine K.J. (2020). A pH sensitive thiolated β-cyclodextrin-modified nanoporous gold for controlled release of doxorubicin. J. Drug Deliv. Sci. Technol..

[14] Ji Q., Qiu L.Y. (2016). Mechanism study of PEGylated polyester and β-cyclodextrin integrated micelles on drug resistance reversal in MRP1-overexpressed HL60/ADR cells. Colloids Surf. B.

[15] Kim C., Jeong D., Kim S., Kim Y., Jung S. (2019). Cyclodextrin functionalized agarose gel with low gelling temperature for controlled drug delivery systems. Carbohydr. Polym..

[16] Zhou Z.S., Guo F., Wang N.R., Meng M., Li G.Y. (2018). Dual pH-sensitive Supramolecular micelles from star-shaped PDMAEMA based on β-cyclodextrin for drug release. Int. J. Biol. Macromol..

[17] Lv J.Q., Liang R.C., Xia Z.H., Li Y., Lv Z.F., Hou D.Z., Yu L.P., Chen G., Liu Y., Yang F. (2019). Synthesis and characterization of Amphiphilic star-shaped copolymers based on β-cyclodextrin for micelles drug delivery. Drug Dev. Ind. Pharm..

[18] Ramesh K., Balavigneswaran C.K., Siboro S.A.P., Muthuvijayan V., Lim K.T. (2021). Synthesis of cyclodextrin-derived star poly(N-vinylpyrrolidone)/poly(lactic-co-glycolide) supramolecular micelles via host-guest interaction for delivery of doxorubicin. Polymer.

[19] Gao Y.R., Li G.Y., Zhou Z.S., Gao L.L., Tao Q. (2017). Sensitive complex micelles based on host-guest recognition from chitosan-graft-β-cyclodextrin for drug release. Int. J. Biol. Macrom..

[20] Poudel A.J., He F., Huang L.X., Xiao L., Yang G. (2018). Supramolecular hydrogels based on poly (ethylene glycol)-poly (lactic acid) block copolymer micelles and alpha-cyclodextrin for potential injectable drug delivery system. Carbohydr. Polym..

[21] Huang X.J., Xiao Y., Lang M.D. (2011). Self-assembly of pH-sensitive mixed micelles based on linear and star copolymers for drug delivery. J. Colloid Interface Sci..

[22] Jain V., Jain S., Mahajan S.C. (2015). Nanomedicines based drug delivery systems for anticancer targeting and treatment. Cur. Drug Deliv..

[23] Ganta S., Devalapally H., Shahiwala A., Amiji M. (2008). A review of stimuli-responsive nanocarriers for drug and gene delivery. J. Control. Release.

[24] Shang Y.Q., Guo L.X., Wang Z.G. (2019). Tetraphenylsilane Cored Star Shaped Amphiphilic Block Copolymers for pH-Responsive Anticancer Drug Delivery. Macrom. Chem. Phys..

[25] Mao J., Li Y., Wu T., Yuan C.H., Zeng B.R., Xu Y.T., Dai L.Z. (2016). A simple dual-pH responsive prodrug-based polymeric micelles for drug delivery. ACS Appl. Mater. Interfaces.

[26] Dong P.W., Wang X.H., Gu Y.C., Wang Y.J., Wang Y.J., Gong C.Y., Luo F., Guo G., Zhao X., Wei Y.Q. (2010). Self-assembled biodegradable micelles based on star-shaped PCL-*b*-PEG copolymers for chemotherapeutic drug delivery. Colloids Surf. A Physicochem. Eng. Asp..

[27] Arbuck S.G., Christian M.C., Fisherman J.S., Cazenave L.A., Sarosy G., Suffness M., Adams J., Canetta R., Cole K.E., Friedman M.A. (1993). Clinical development of Taxol. J. Natl. Cancer Inst. Monogr..

[28] Wang L., Li H., Ren Y., Zou S., Fang W., Jiang X., Jia L., Li L., Liu X., Yuan X. (2016). Targeting HDAC with a novel inhibitor effectively reverses paclitaxel resistance in non-small cell lung cancer via multiple mechanisms. Cell Death Dis..

[29] Zhong N., Byun H.S., Bittrnan R. (1998). An Improved Synthesis of 6-O-Monotosyi-6-deoxy-β-cyclodextrin. Tetrahedron Lett..

[30] Yoon J.Y., Hong S.Y., Martin K.A., Czarnik A.W. (1995). A General Method for the Synthesis of Cyclodextrinyl Aldehydes and Carboxylic Acids. J. Org. Chem..

[31] Malenkovskaya M.A., Shipilov D.A., Vasyanina L.K., Grachev M.K. (2016). Synthesis of 6-Mono-aldehyde of β-cyclodextrin and imino derivatives on its basis. Rus. J. Gen. Chem..

[32] Xu M.Y., Wu S.Z., Zeng F., Yu C.M. (2010). Cyclodextrin supramolecular complex as a water-soluble ratiometric sensor for ferric ion sensing. Langmuir.

[33] Kim J.H., Li Y., Kim M.S., Kang S.W., Jeong J.H., Lee D.S. (2012). Synthesis and evaluation of biotin-conjugated pH-responsive polymeric micelles as drug carriers. Int. J. Pharm..

[34] Sun T.B., Jin Y., Qi R., Peng S.J., Fan B.Z. (2013). Oxidation responsive monocleavable amphiphilic di-block polymer micelles labeled with a single diselenide. Polym. Chem..

[35] Lin W.J., Yang C.F., Xue Z.L., Huang Y.H., Luo H.S., Zu X.H., Zhang L.J., Yi G.B. (2018). Controlled construction of gold nanoparticles in situ from β-cyclodextrin based unimolecular micelles for in vitro computed tomography imaging. J. Colloid Interface Sci..

[36] Mastromatteo M., Conte A., Del Nobile M.A. (2010). Advances in controlled release devices for food packaging applications. Trends Food Sci. Tech..

