# Supplementary material for: Preparation and Release of pH-Sensitive β-Cyclodextrin Derivative Micelles Loaded with Paclitaxel
Source: Polymers (Basel). 2022 Jun 18;14(12):2482. doi: 10.3390/polym14122482 (PMC9227914; doi:10.3390/polym14122482)
Supplement: Supplementary file 1 [file polymers-14-02482-s001.zip › polymers-1750314-supplementary.pdf]

Supplementary Materials

Preparation and release of pH-sensitive β-cyclodextrin derivative micelles loaded with paclitaxel

Meirong Zhao [1], Weiwei Jiang [1], Xinrong Xie, Yogini Jaiswal, Leonard Williams, Mei Wei, Ying Mo, Yifu Guan * and Hua Yang *

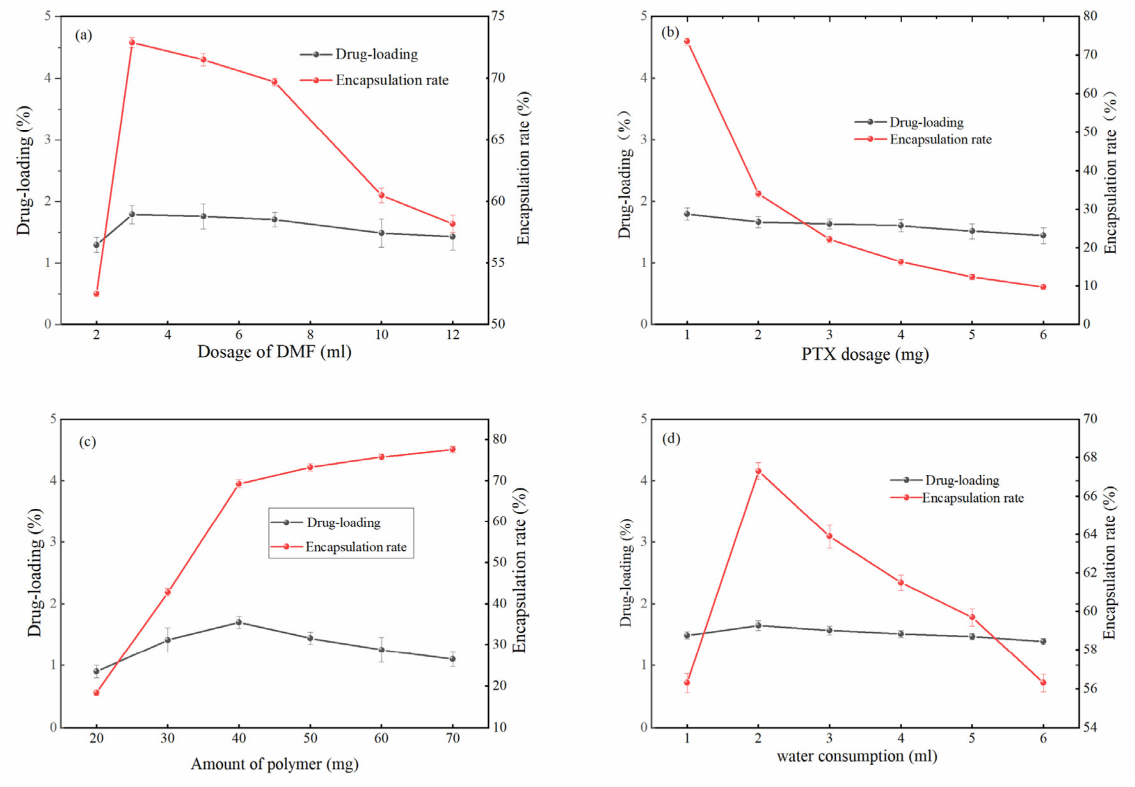

Figure S1. Results of the effect of the quantity of DMF (**a**), PTX (**b**), polymer (**c**), and water (**d**) on PTX loading.

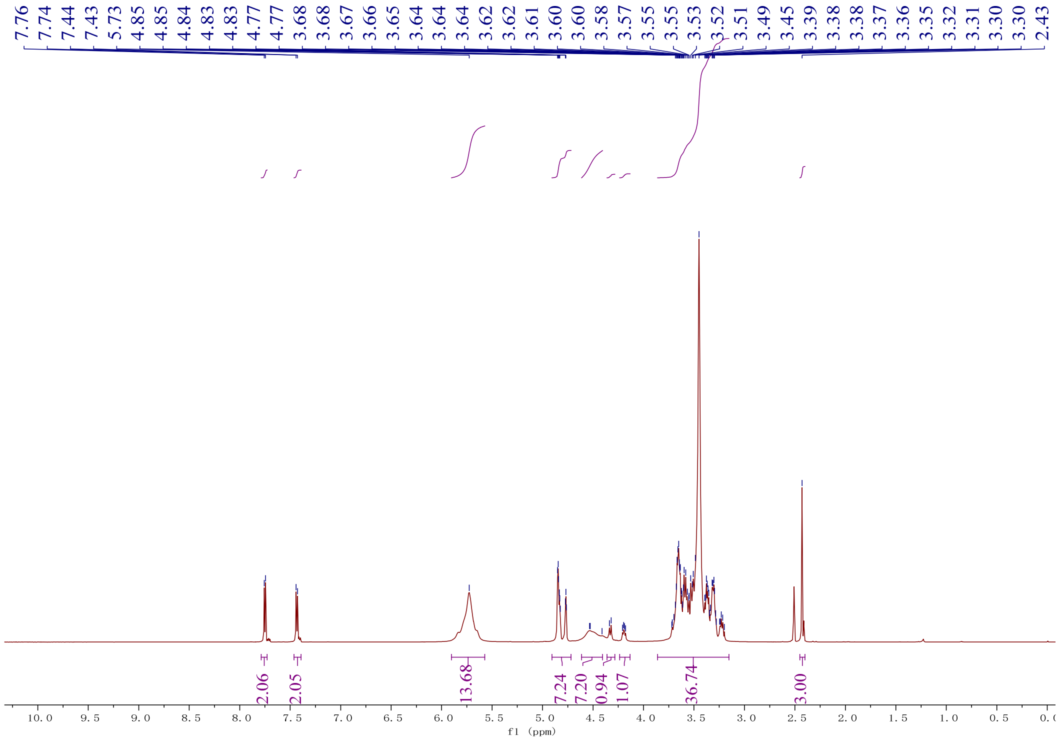

Figure S2. ^{1}H NMR of 6-OTs-β-CD

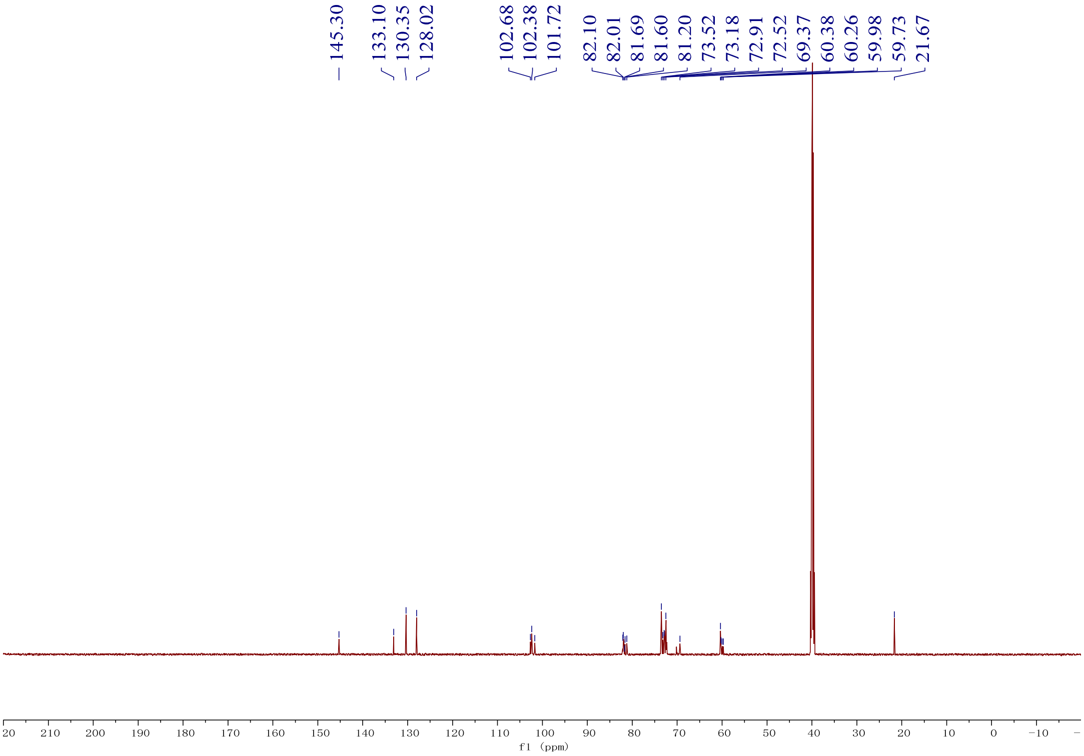

Figure S3. ^{13}C NMR of 6-OTs-β-CD

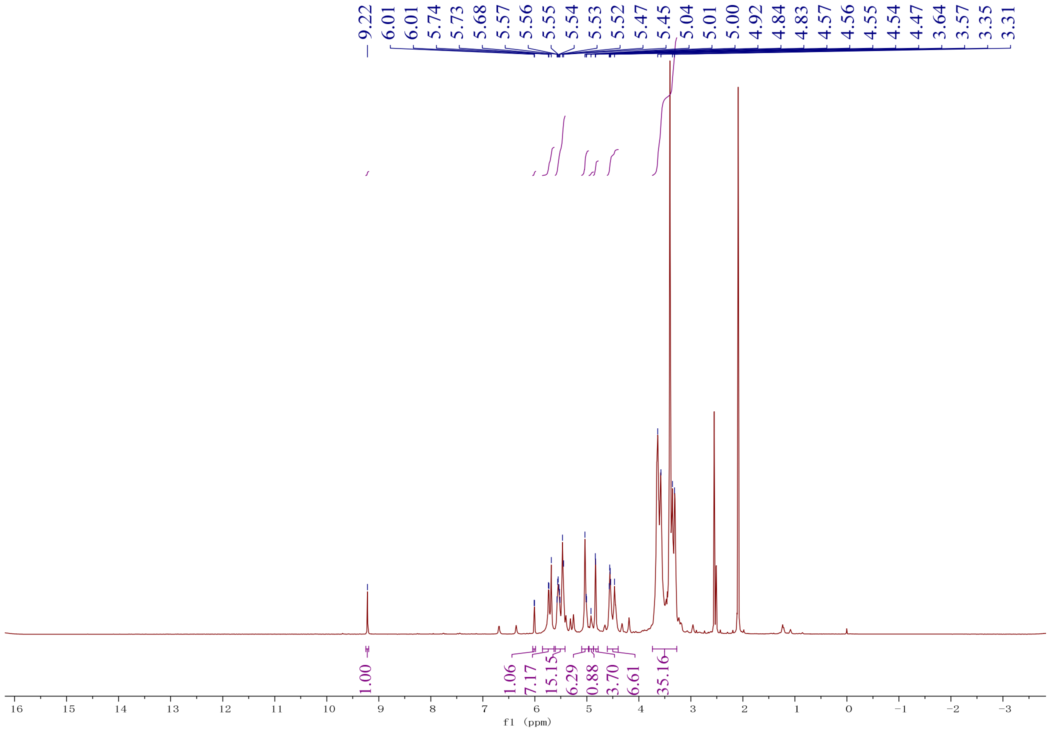

Figure S4. ^{1}H NMR of 6-CHO-β-CD

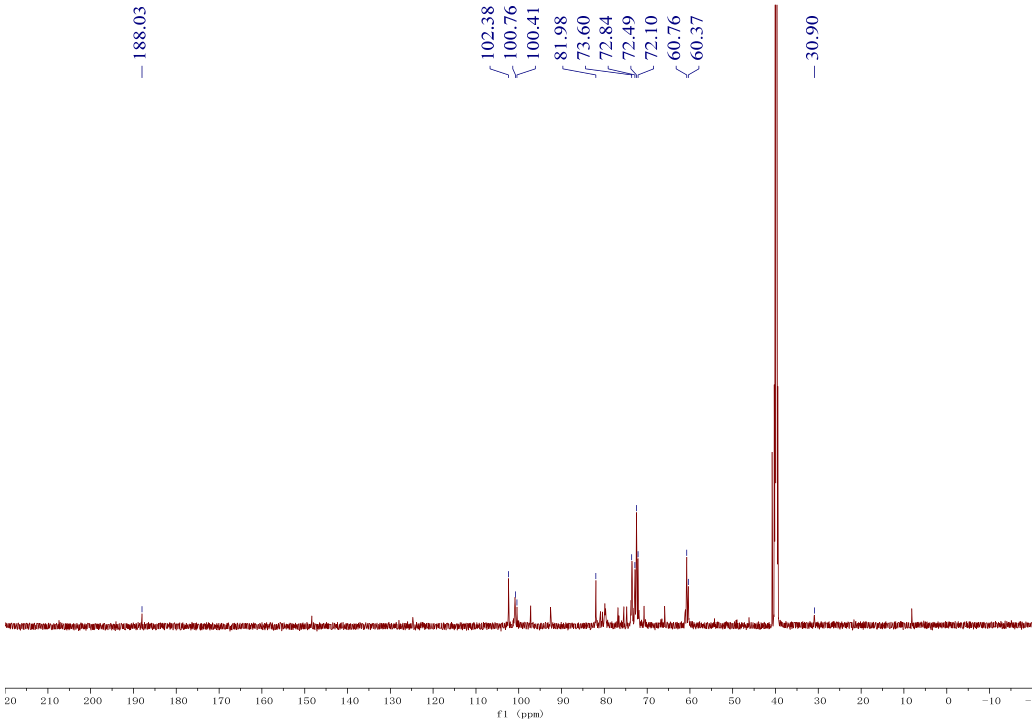

Figure S5. ^{13}C NMR of 6-CHO-β-CD

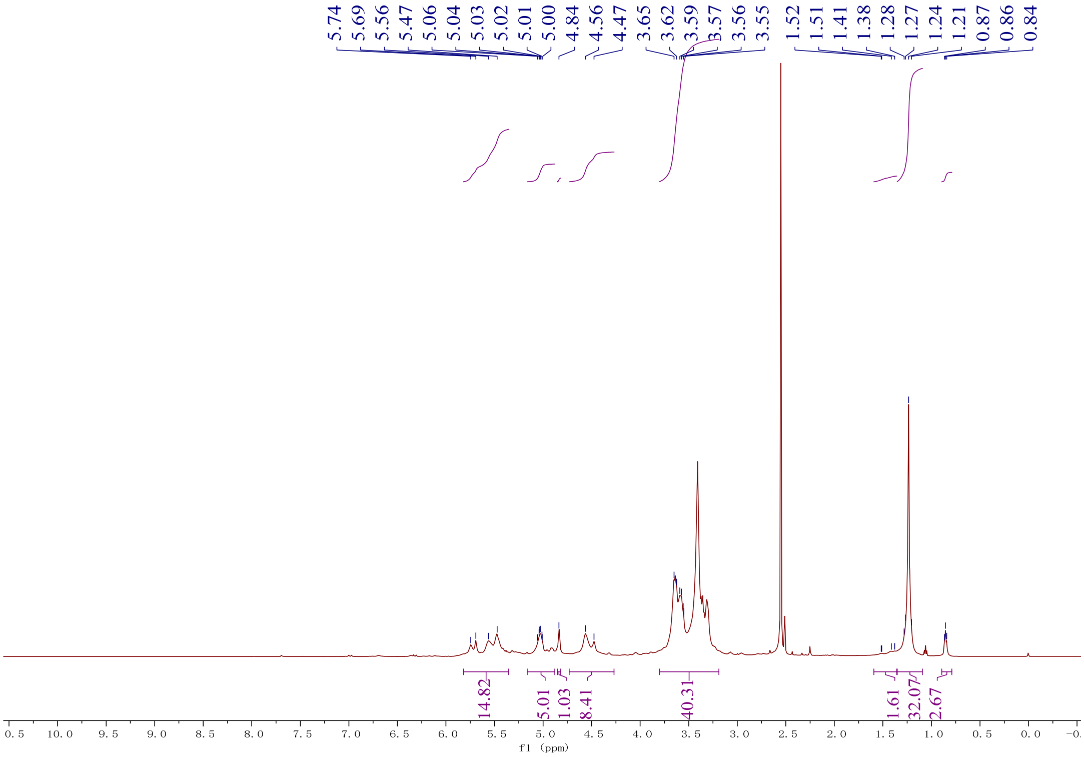

Figure S6. ^{1}H NMR of β-CD-N-ODMA

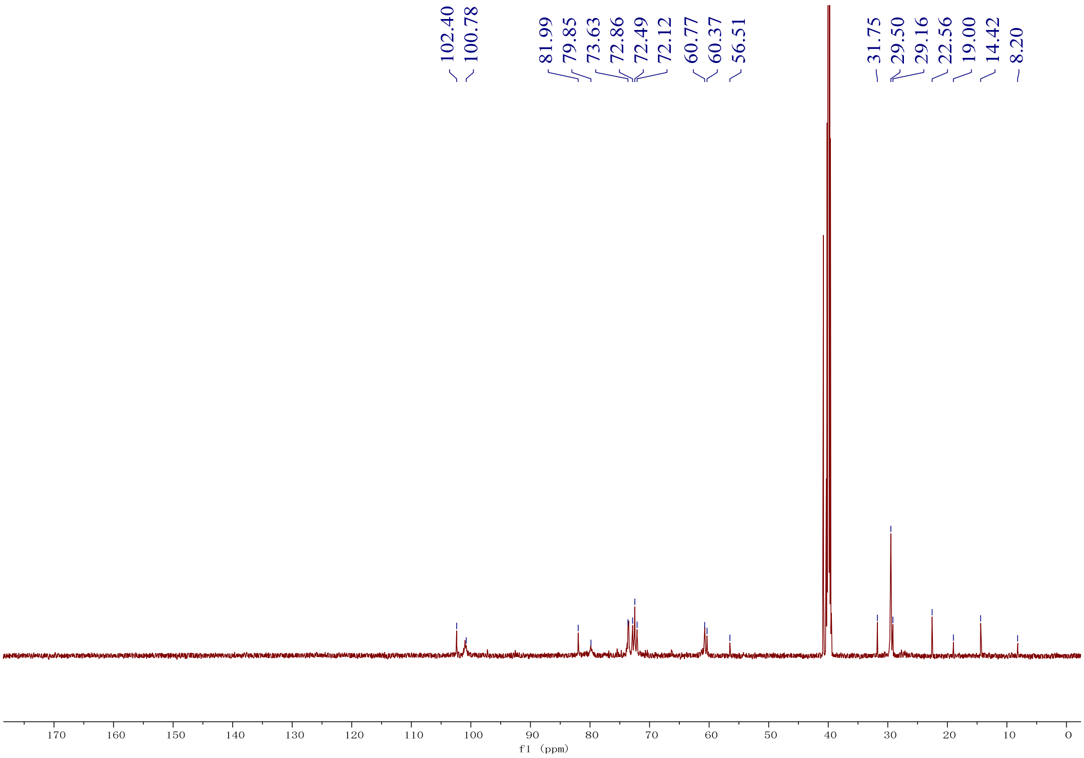

Figure S7. ^{13}C NMR of β-CD-N-ODMA
